# *Centella asiatica* Prevents Increase of Hippocampal Tumor Necrosis Factor-α Independently of Its Effect on Brain-Derived Neurotrophic Factor in Rat Model of Chronic Stress

**DOI:** 10.1155/2019/2649281

**Published:** 2019-03-06

**Authors:** Mawaddah Ar Rochmah, Ika Murti Harini, Dian Eurike Septyaningtrias, Dwi Cahyani Ratna Sari, Rina Susilowati

**Affiliations:** ^1^Department of Anatomy, Faculty of Medicine, Public Health, and Nursing, Universitas Gadjah Mada, Yogyakarta 55281, Indonesia; ^2^Department of Neurology, Faculty of Medicine, Public Health, and Nursing, Universitas Gadjah Mada, Yogyakarta 55281, Indonesia; ^3^Department of Histology and Cell Biology, Faculty of Medicine, Public Health, and Nursing, Universitas Gadjah Mada, Yogyakarta 55281, Indonesia; ^4^Department of Histology, Faculty of Medicine, Universitas Jenderal Soedirman, Purwokerto, Central Java 53112, Indonesia

## Abstract

*Centella asiatica* ameliorates memory impairment and induces expression of hippocampal brain-derived neurotropic factor (BDNF) in chronically stressed rats. The relationship between the anti-inflammatory effect of* Centella asiatica *on hippocampal BDNF and the involvement of sirtuin-1, a BDNF expression regulator, in neuroprotective mechanisms of* Centella asiatica* warrants an investigation. We investigated the effect of* Centella asiatica* ethanolic extracts (CA) on TNF-*α*, IL-10, and SIRT1 levels and whether these predicted BDNF expression in rat hippocampus after chronic stress. For the experiments, thirty male rats (*Sprague Dawley*) were divided into six groups: nonstressed-control, stressed-control, nonstressed +CA 300mg/kg/d, stressed +CA 150 mg/kg/d, stressed +CA 300 mg/kg/d, and stressed +CA 600 mg/kg/d. On day 28, rats were sacrificed and hippocampus was dissected out. Hippocampal TNF-*α*, IL-10, SIRT1, and BDNF were measured by enzyme-linked immunosorbent assay. Hippocampal TNF-*α* level was significantly higher in the stressed-control compared to nonstressed-control groups. Across all stress conditions, rats receiving the highest dose of CA had the lowest mean TNF-*α* and highest mean BDNF. There were no significant differences in IL-10 and SIRT1 levels between groups. Hippocampal TNF-*α* did not predict hippocampal BDNF in a regression analysis. In conclusion, lower TNF-*α* and higher BDNF in the hippocampus support the hypothesis that these factors independently contribute to* Centella asiatica'*s neuroprotective effect in chronically stressed rats.

## 1. Introduction

*Centella asiatica* (L.) urban, known as Pegagan in Indonesia, is a medicinal plant of the* Umbelliferae* (*Apiaceae*) family, widely grown in Asian countries as well as in South Africa and Eastern Europe [1]. This medicinal plant has primary active compounds in saponin (triterpenoid) forms, such as asiaticocide, asiatic acid, madecassoside, and muriatic acid [1].* Centella asiatica* (*C. asiatica*), along with its active compounds, have been widely studied and reported to possess many properties, such as neuroprotective [2], antinociceptive [3], antioxidant, and antihyperlipidemic [4] as well as anticancer [5]. The neuroprotective property of* C. asiatica* makes this plant as a potential candidate for treatment against cognitive decline and other nervous system degeneration [6].

Recently, we reported that* C. asiatica * leaf ethanolic extract (CA) prevents the decrease of hippocampal brain-derived neurotrophic factor (BDNF) concentration in hippocampus upon chronic stress [7]. Luo et al. similarly reported upregulation of hippocampal BDNF in chronic stress in mice receiving asiaticocide [8]. BDNF is an important mediator of neuroplasticity, i.e., neurogenesis and neural remodeling, which protects nervous system from detrimental effects of chronic stress [9, 10]. The neuroprotective effect of BDNF is regulated by cAMP-response element binding protein (CREB) complex which is activated by sirtuin 1 (SIRT1), a nicotinamide-adenine dinucleotide- (NAD-) dependent protein deacetylase [11]. Because there are other pathways in inducing BDNF expression [12, 13], whether SIRT1 is involved in the effect of* C. asiatica* on hippocampal BDNF needs to be elucidated.

Neuroinflammation is implicated in many neurological disorders including memory deficit due to chronic stress [14]. In the hippocampus of chronically stressed rats, expression of proinflammatory cytokines such as tumor necrosis factor-*α* (TNF-*α*) is reported to be upregulated while the expression of anti-inflammatory cytokines such as interleukin 10 (IL-10) is suppressed [15–18]. The altered cytokine expression has been associated with impaired memory [14, 19]. The effect of stress-induced inflammation on the impairment of memory may be mediated by BDNF, as it has been suggested in recent reviews [20, 21].

*C. asiatica* and its active compounds are also known to have anti-inflammatory effects [22, 23]. Anti-inflammatory mechanism of asiaticoside has been proven to attenuate memory impairment in a mice model of cerebral ischemia [24]. These effects may be associated with the inhibition of proinflammatory mediators, including TNF-*α* [25]. Suppressing inflammation by inhibiting TNF-*α* expression using medicinal plants has been shown to improve memory deficits and depression [26, 27]. Aqueous extract of* C. asiatica* prevents the increase of hippocampal TNF-*α* in diabetic rats [28]. However, there is limited data on the effect of* C. asiatica* on hippocampal TNF-*α* and IL-10 of chronically stressed rat. Whether* C. asiatica*'s anti-inflammatory effect is correlated with its BDNF-dependent neuroprotective pathway* in vivo* is also unknown.

This study aimed to investigate the effect of CA on hippocampal TNF-*α*, IL-10, and SIRT1 as well as their possible involvement in inducing hippocampal BDNF concentration in rat model of chronic stress. Repetitive electrical foot shock was chosen for this study because this model was previously used in the report on* C. asiatica* effect in preventing memory impairment and the reduction of BDNF concentration upon chronic stress in rats [7]. In this study, the hippocampal tissue was directly obtained after 28 days of repetitive electrical foot shock and CA administration.

## 2. Material and Method

### 2.1. Ethanolic Extracts of* Centella asiatica* Leaf

The* C. asiatica* leaf was purchased from CV. Merapi Farma Herbs, Sleman, Yogyakarta, Indonesia. Taxonomic identification of the plant material was confirmed by a botanist at the Department of Botany, Faculty of Biology, Universitas Gadjah Mada, Indonesia. The dried* C. asiatica* leaves were crushed into fine powder and CA was prepared in 70% ethanol at Laboratorium Penelitian dan Pengujian Terpadu Universitas Gadjah Mada. Each gram of dried* C. asiatica* leaf could yield 194.37 mg of ethanolic extract. The extract was stored at 4°C until further use. Thin layer chromatography showed 0.158% w/w of asiaticoside content within the CA.

### 2.2. Experiments on Rat Model of Chronic Stress

All experimental procedures were approved by Medical and Health Research Ethics Committee Faculty of Medicine Universitas Gadjah Mada no Ref KF/FK/813/EC. Thirty rats (one-month-old male Sprague Dawley) were used in this study. The rats were kept in cages consisted of two to three rats per cage with water and food* ad libitum*. Room temperature was 21°C with 50-60% humidity and light-dark cycle of 12:12 hours. The rats were divided into six groups: Group A (nonstressed-control), Group B (stressed-control), Group C (nonstressed + CA 300mg/kg/d), Group D (stressed + CA 150 mg/kg/d), Group E (stressed + CA 300 mg/kg/d), and Group F (stress + CA 600 mg/kg/d). During the experiment, the rats were weighed on days 0, 7, 14, and 28. Weight change was calculated, and body weight reduction compared to nonstressed-control group was observed as a sign of stress [29].

Daily administration of CA was done orally using a gastric tube. Electrical stress was performed at 30 minutes after CA administration in a plexiglas shock box, of 48 cm length, 24 cm width, and 32 cm height, equipped with a wired grid floor, designed according to previous study [30]. This box is connected with ampere-meter to measure the electric current, a voltmeter to measure the electrical voltage and stabilizer to stabilize the voltage. The conditions applied was current of 0.8 mA, 5 seconds each, three times per minutes for a total of 10 minutes. On day 28, rats were sacrificed by decapitation and hippocampus regions were dissected out for protein extraction.

### 2.3. Hippocampal Protein Extraction and Measurement of TNF-*α*, IL-10, SIRT1, and BDNF

Protein extraction from fresh rat hippocampus was performed using PRO-Prep Protein Extraction Solution (Intron cat no 17081, Intron Biotechnology Inc, Korea) according to manufacturer guideline and stored at –80°C until analysis. The enzyme-linked immunosorbent assay (ELISA) was used to quantify TNF-*α* (Sigma RAB0480-1KT, Sigma-Aldrich Co. MO, USA), IL-10 (Sigma RAB0247-1KT, Sigma-Aldrich Co. MO, USA), SIRT1 (USCN E94912Ra, Uscn Life Science Inc., GA, USA), and BDNF (Boster EK0308 size 96T, Boster-Bio, CA, USA). Concentrations of TNF-*α*, IL-10, SIRT1 and BDNF were derived from optical density (OD) values using standard curves. Each measurement was performed in duplicate.

### 2.4. Statistical Analysis

The homogeneity of the data was tested using the Shapiro-Wilk normality test. The data were presented as mean (95% confidence interval (CI)). The difference between groups was analyzed by one-way analysis of variance (ANOVA) followed by Tukey's post hoc analysis. Linear regression was used to analyze the concentration of hippocampal TNF-*α*, IL-10, and SIRT1 in predicting the concentration of hippocampal BDNF. All analyses were performed using GraphPad Prism version 4 (GraphPad Software Inc., La Jolla, CA, USA). All tests were two-tailed, and* p*<0.05 was considered statistically significant.

## 3. Results

### 3.1. Effect of CA on Hippocampal Concentration of TNF-*α*, IL-10, SIRT1, and BDNF

**Figure 1:**
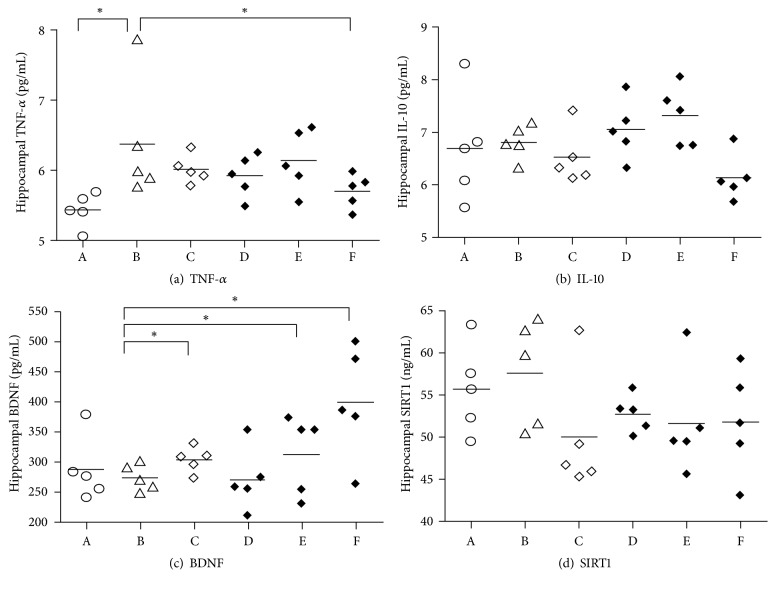
*The concentration of hippocampal tumor necrosis factor-α (TNF-α) (a), interleukin 10 (IL-10) (b), brain-derived neurotrophic factor (BDNF) (c), and sirtuin-1 (SIRT-1) (d)*. Horizontal axis represents rats' groups: Groups A (nonstressed-control), B (stressed-control) C (nonstressed + CA 300mg/kg/d), D (stressed + CA 150 mg/kg/d), E (stressed + CA 300 mg/kg/d), and F (stress + CA 600 mg/kg/d). *∗*ANOVA followed by Tukey post hoc test <0.05.

TNF-*α* (pg/mL) ranged from 5.05 to 7.86. TNF-*α* was significantly higher in the stressed-control group (F(5,24) = 2.741; p = 0.043). The group receiving the highest dose of CA (Group F; 5.70 (95%CI 5.40;6.00) had the lowest concentration of hippocampal TNF-*α* compared to other groups receiving stress, and this was significantly lower than the stressed-control group (Group B; 6.37 (95% CI 5.30;7.44). There was no significant difference between mean TNF-*α* in groups receiving lower doses of CA (Groups C, D, and E) and the stressed-control group (Figure 1(a)).

Hippocampal IL-10 did not significantly decrease in stressed-control (Group B) compared to nonstressed-control rats (Group A) (F(5,24) = 2.227; p = 0.085). Administration of CA in all three doses (Groups C, D, E, and F) did not significantly alter mean of hippocampal IL-10 (Figure 1(b)). Similarly for SIRT1, there were no significant differences between groups (F(5,24) = 1.213; p = 0.333) (Figure 1(d)).

There was a significant difference in hippocampal BDNF concentration between groups (F(5,24) = 3.503; p = 0.016). Although hippocampal BDNF was lower in the stressed-control group (Group B; 274.50; 95% CI 247.31;301.69) compared to nonstressed-control group (Group A; 287.5, 95% CI 220.68;354.32) (Figure 1(c)), this was not statistically significant. Hippocampal BDNF was higher in the 300 and 600 mg/kg CA groups. The rats receiving the highest dose of CA (Group F; 600 mg/kg) had the highest BDNF (400; 95%CI 284.44; 515.56). The concentration of BDNF in Group F receiving CA 600 mg/kg was not significantly different to the stressed (Group E) and unstressed (Group C) groups that received CA 300 mg/kg.

**Figure 2:**
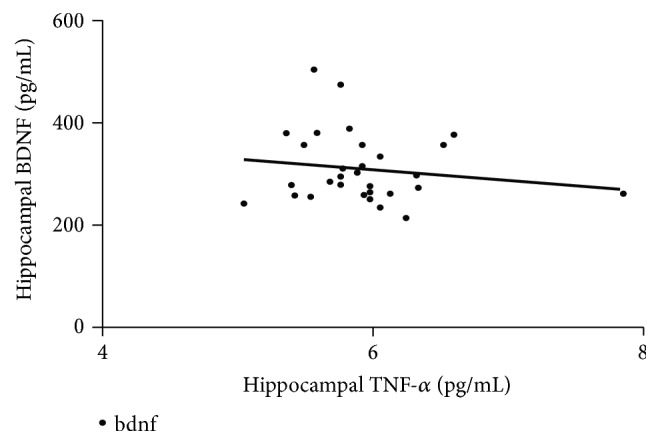
*Linear regression analysis of hippocampal TNF-α and BDNF*. Horizontal axis represents hippocampal TNF-*α* as an independent variable and vertical axis represents hippocampal BDNF as a dependent variable (P>0.05).

Linear regression was performed to analyze whether increased BDNF concentration could be predicted by the concentration of TNF-*α*, IL-10, and SIRT1.  Figure 2 showed the scatter plot for hippocampal TNF-*α* as predictor of hippocampal BDNF. However, none of them significantly predicted hippocampal BDNF (p>0.05). Pearson correlation analysis also could not find any significant correlation between variables.

### 3.2. Effect of CA on Body Weight

All rats survived until the termination day. There was no significant difference in mean body weight between groups at baseline. All rats gained weight during the 28 day experimental phase, range 32 to 120 g. Differences in mean weight gain between groups were small (Table 1) and not statistically significant.

## 4. Discussion

Hippocampal TNF-*α* is higher in rats which received chronic repetitive electrical foot shock, supporting previous report which used a restraint stress model [31]. Increased proinflammatory cytokines in hippocampus have been reported in other rat model such as ischemic brain [24] and diabetes [28]. However, to our knowledge, this is the first report on hippocampal TNF-*α* in a rat model administering chronic repetitive electrical foot shock. Our data supports the use of hippocampal TNF-*α* as a more specific marker of chronic stress in rodents than reduced body weight. Discrepancy in previous studies regarding reduced and nonreduced body weight in chronically stressed rats suggests that reduced body weight cannot be used as a single parameter to quantify chronic stress in rodents [29, 32–34]. The plasma concentration of TNF-*α* as well as other inflammatory cytokines increases under stressful condition such as restraint stress [35]. Therefore, it would be feasible to use serial plasma TNF-*α* for evaluating stress response in rodents to chronic stress paradigms, such as what has been shown for serum BDNF [36].

Our data showed no significant difference in hippocampal IL-10 between groups. Therefore, we could not provide support for the hypothesis that CA treatment might alleviate stress-induced diminution of hippocampal IL-10. There are reports that serum IL-10 increases, not decreases, in rodents subjected to chronic stress [35] and maternal deprivation [19]. This suggests that some cytokines may have antagonistic effect, i.e., pro- and anti-inflammatory effect, in accordance with the site and time points. Acute and subacute administrations of cytokines have been shown to induce different changes toward neuroprotection or neurodegeneration [37]. However, in this study, there was no significant association between hippocampal IL-10, the chronic stress condition, and CA administration which suggests IL-10 might not be strongly involved in the hippocampal stress response.

The CA treatment groups showed higher hippocampal BDNF compared to stressed-control group. This result supports our previous findings that CA increases BDNF concentration in serum and hippocampal tissue of chronically stressed rats postwater maze test [7, 36]. Since water maze test takes at least 1 week to perform, there is a time gap between the last foot shock and CA administration with tissue sampling. This study provides data of CA effect on BDNF concentration directly after the last electrical foot shock and CA administration. Generally, the hippocampal BDNF in this study had higher concentration compared to hippocampal BDNF after water maze test [7]. There are two possibilities that may lead to this result: first, BDNF concentration decreases after the termination of CA administration; second, the water maze test may provide stressful environment that leads to decreased hippocampal BDNF expression. This result also supports the finding that CA ameliorates the stress effect in hippocampal BDNF in a dose-dependent manner, with the highest dose (600mg/kg/day) of CA displaying the highest increase of hippocampal BDNF. Considering the asiaticoside content in the extract is only 0.158% w/w extract, the dose used in this study is lower compared to previous report on the asiaticoside anti-inflammatory effect in cerebral ischemia mice model [24].

We found no correlation between hippocampal BDNF and SIRT1 concentration. Furthermore, no significant differences in mean hippocampal SIRT1 among the experimental groups could be observed. Therefore, the result suggests that SIRT1 and their subsequent targets such as CREB may not be directly involved in the mechanism of CA effect on hippocampal BDNF. Similar results were reported in a study of the effect of another medicinal plant,* Boswellia sp*., that failed to provide evidence of CREB involvement in Boswellia's effect on BDNF [38].

The present study did not find an association between hippocampal TNF-*α* and hippocampal BDNF. This result does not support one report about the ability of infliximab, a TNF-*α* inhibitor, in preventing cognitive decline and maintaining hippocampal BDNF in an unpredictable chronic mild stress rat model [14]. An in vitro study reported that* C. asiatica* induced both BDNF and TNF-*α* in cultured neuronal cells [39], while in this study, CA administration induced higher BDNF and lower TNF-*α* in the hippocampus of chronically stressed rats. One possible explanation is TNF-*α* may directly cause excitotoxicity [40] which in turn prevent a detectable change in other signaling pathways. Because* C. asiatica*'s anti-inflammatory properties are not restricted to brain [41] and peripheral inflammatory signals may contribute in alteration of gene expression in the brain [42], hippocampal BDNF expression may not be directly correlated with hippocampal TNF-*α*.

In this study, we investigated hippocampal TNF-*α*, IL-10, SIRT1, and BDNF data at the protein level to provide a precise description of their activity in rat hippocampus. However, no positive correlation between variables could be observed. We consider that the small sample size in each group might be the limitation of this study. Therefore, for future investigation, we suggest additional sample size and measurement of gene expression at mRNA level that may provide a more sensitive detection to complete the transcription data of each observed variable.

## 5. Conclusion

In conclusion, we showed that high dose of CA consistently induced higher hippocampal BDNF in rat model of chronic stress. New data of CA ameliorating the increase of hippocampal TNF-*α* in chronic repetitive electrical foot shock model in rat was also provided. Therefore, this study strengthens the evidence that* C. asiatica* has properties that moderate the effects of chronic stress in the hippocampus. Because no significant direct correlation was found between hippocampal BDNF with SIRT1, TNF-*α*, and IL-10, new direction should be pursued in order to better understand the mechanism of* C. asiatica* in preventing BDNF reduction upon chronic stress.

## Figures and Tables

**Table 1:** Weight gain in rat model of chronic stress (28 days of electrical shock).

groups	mean	95% confidence interval		p value
		lower	upper	
A	70	52.44	87.56	0.999
B	66	43.44	88.56	
C	68	44.12	91.88	
D	68	45.79	90.21	
E	68	54.40	81.60	
F	70	32.75	107.25	

## Data Availability

The data and materials supporting the conclusion of this research article are included within the article.
